# Dataset of a numerical model of a cold-formed, C-shaped section in compression according to EN 1993-1-14

**DOI:** 10.1016/j.dib.2024.110097

**Published:** 2024-01-24

**Authors:** Dieter Ungermann, Tim Lemański, Bettina Brune

**Affiliations:** Chair of Steel Construction, TU Dortmund University, August-Schmidt-Str. 6, 44227 Dortmund, Germany

**Keywords:** Thin-walled cold-formed steel sections, FEM, GMNIA, ANSYS, Flexural torsional buckling, Experimental data, Imperfections

## Abstract

Numerical simulations have gained increasing importance in civil engineering in the recent past. The potential for research on the one hand and for practical application on the other hand is great. EN 1993-1-14 was developed to support users in the generation of numerical models, to provide security in the procedure and to achieve comparable results across the board. The accompanying publication of application examples is of extraordinary assistance. The data set presented in this article was also used to generate the benchmark examples No. 5 of Technical Report EN/TR 1993-1-141:2023 (E)_v2023-06 on EN 1993-1-14. Based on the data, the procedure for validating a numerical model on experimental component tests can be reproduced using a series of tests on cold-formed, C-shaped columns in axial compression. In this article, the measured imperfections of the test specimens and the recorded measured values during the test are first discussed in detail. The corresponding data are provided. Subsequently, the numerical models are provided and described as input files in the program language ANSYS Parametric Design Language (APDL). The entire data set can be used as an aid to generate numerical models according to the specifications of EN 1993-1-14.

Specifications Table

**Table:** 

Subject	Engineering
Specific subject area	Validation of a numerical model according to EN 1993-1-4 for the simulation of compression tests of cold-formed steel sections
Data format	Raw, Analyzed
Type of data	Tables, Charts, ANSYS input files
Data collection	From the experimental tests, strains were recorded via strain gauges (FLAB-6-11-3LJCT-F from TML) and deformations via linear variable differential transformers (WA100 from HBM). Machine displacement and force were recorded by the machine control. Data acquisition was performed using CATMAN software. A UPM 60 multi-point measuring device was used as the interface between the machine and the measurement equipment and the computer. Numerical data were generated via ANSYS software (version 2023 R2) on a Dell OptiPlex 7050. EXCEL (Office 2016) was used to generate the comparative diagrams. The material test certificate with the material properties of the steels used was made available to the researchers.
Data source location	TU Dortmund University, Chair of Steel Construction, August-Schmidt-Str. 6, 44227 Dortmund, Germany TU Dortmund University, Institute of Building Research, Baroper Str. 299, 44227 Dortmund, Germany
Data accessibility	Repository name: Mendeley Data Data identification number DOI:10.17632/4342867j9g.1 Direct URL to data: https://data.mendeley.com/datasets/4342867j9g/1

## 1 Value of the Data

• The data presented in this article describe the standard-compliant procedure for creating a numerical model according to the specifications of the new EN 1993-1-14 [1]. The application of EN 1993-1-14 is explained by means of the simulation of a compression test of a column and can be fully understood by providing the input files of the numerical model.
• Any user can use this data set as a basic template or as an aid in generating their own numerical model for the simulation of cold-formed steel columns in compression.
• This contribution helps to advance the establishment of EN 1993-1-14, which will be available in the future.

## 2 Background

The data set presented in the following was created as part of the FOSTA research project P1328 ``Future viability of cold-formed steel sections in building structures'' [2], in which both experimental and numerical investigations were carried out on thin-walled, cold-formed steel sections. The data presented in this article have already been processed for the preparation of the Technical Report CEN/TR 1993-1-141:2023 (E)_v2023-06 on EN 1993-1-14 [3]. For this purpose, component tests were selected from the research project for which a standard-compliant benchmark example was created for modeling a thin-walled, cold-formed steel section in compression with stability failure due to flexural torsional buckling. This is Example 5 (Annex A5). The results of the numerical simulations are also interpreted there.

The data provided in this article extends the benchmark example with additional graphics, evaluations and, in particular, numerical input files in ANSYS Parametric Design Language (APDL). Thus, this data set serves to significantly extend the assistance provided by the Technical Report for the application of EN 1993-1-14 for users.

## 3 Data Description

### 3.1 General structure

The data set in the repository is divided into three folders: ``FE input files'', ``FE Data'' and ``Experimental Data and Comparison to FE''.

### 3.2 Folder ``FE input file''

The folder ``FE input files'' contains the ANSYS input files as mac files. The mac files contain the commands in the APDL program language to generate the numerical model. All files contain the definition of parameters (test specimen length, cross-section dimensions, applied load, etc., as well as the parameters according to Table 1), the creation of the component geometry, the meshing of the geometry, the assignment of the material parameters and the definition of the boundary conditions. The file *01_Buckling.mac* contains additional the commands to perform an elastic buckling analysis. File *02_Precurvature.mac* contains the definition of the boundary conditions to generate a sinusoidal precurvature over the length of the specimen. The file *03_DTB_Imperfection.mac* is structured analogously to the previous files. It contains the definition of the boundary conditions for the generation of the sinusoidal imperfection of the lips corresponding to the stability phenomena distortional buckling. The file *04_Bearing_Load.mac* contains an implementation of the imperfections and the final determination of the ultimate load as the maximum of the recorded load-deformation curve. All commands in the input files are commented.

**Table 1:** Parameters that can be adjusted in the input files to perform the simulations listed in this article.

Parameter	Meaning	Unit	Examined values	Relevant for input file
*etG*	Element size	mm	1.25, 2.5, 5, 10, 20, 40	all
*etTyp*	Element type	-	181=Shell181 281=Shell281	all
*t*	Thickness	mm	2.88, 2.89, 3.00	all
*Komb*	Combination of imperfections	-	1-43 according to Table 4, Table 5, Table 6, Table 7, Table 8	*03_DTB_Imperfektion* *04_Bearring_Load*
*Vari_Prec*	Number of Precurvature-Type	-	1=L/1000 in z-direction 2=-L/1000 in y-direction 3=L/1000 in y-direction 4-6=measured precurvature of the test specimen 1-3	*02_Precurvature*
*Sol_Meth*	Selection of the solution algorithm	-	1=Newton-Raphson technique 2=arc length technique	*04_Bearing_Load*

Table 1 summarizes the parameters used in the mac files, which have to be adjusted for the created calculations. These include the element size *etG*, the element type *etType*, the sheet thickness of the section *t*, the combination of imperfections to be applied *Komb* and the solution algorithm *Sol_Meth*. By entering the appropriate parameters, each simulation presented in this article can be reproduced.

### 3.3 Folder ``FE Data''

The folder “FE Data” contains the exported output files that are created when the input file *04_Bearing_Load.mac* is executed.

In the files *Strain_Gauges_Komb.txt* (with *Komb* the number of imperfection combination) the time steps *time*, per time step the applied force *Machine-Force*, the displacement at the point of load application *Machine-Displacement*, the number of elements *elements* and the strains and stresses at the positions where the strain gauges (SG) are located in the test *North_Strain, North_Stress, East_Strain, East_Stress, South_Strain, South_Stress* are documented.

The files *LVDT_Komb.txt* contain the time steps *time* and the displacements at the positions of the linear variable differential transformers (LVDT) *North_Displacement, East_Displacement* and *South_Displacement*.

The results of a sensitivity study for the mesh density are also stored as .txt files in this folder and are described as *LVDT_13_etG_etTyp.txt* (with *etG* and *etTyp* according to Table 1) or *Strain_Gauges_13_etG_etTyp.txt*. For columns in which there is only “*0.00000000*” in each row, the evaluation point for the LVDT or SG was not available due to the coarse meshing.

All values exported from the numerical simulations for the different combinations of imperfections are additionally summarized in the Excel file *FE_Output.xlsx*. The first sheet “*Overview*” provides an overview of the parameters that must be set in the ANSYS input files in order to generate the corresponding results. The sheet also contains information about which input files need to be run. The imperfections applied for each simulation are also summarized. The other sheets of the Excel file contain the results of the simulations with different configurations of imperfections. The exported data for *time, machine force, machine displacement, elements, North_Strain, North_Stress, East_Strain, East_Stress, South_Strain, South_Stress, North_Displacement, East_Displacement, South_Displacement* can be found for each simulation. In addition, each Excel sheet contains the maximum ultimate load *R_dm_* or *R_FE_* of the respective simulation.

The Excel file *Evaluation_Mesh_study.xlsx* contains the evaluation of the parameter study on the element size including the extrapolated values determined in Excel.

### 3.4 Folder ``Experimental Data and Comparison to FE''

The folder ``Experimental Data and Comparison to FE'' contains three Excel files. Each Excel file contains the data from the results of the three experimental test (*Test Specimen 1.xlsx, Test Specimen 2.xlsx, Test Specimen 3.xlsx*) and the results of the numerical simulation using the imperfections measured in the experiment (*Komb* 41-43).

Each Excel file consists of 10 sheets. The ``*Imperfection*'' sheet contains the measured local and global imperfections of the test specimens and is evaluated graphically using diagrams. All raw data collected during the test can be found in the spreadsheet ``*Data raw*'', the data modified for charting can be found in the spreadsheet ``*Data modified*'', and the charts for evaluation in the spreadsheets ``*Force-LVDT*'' and ``*Force-SG*''. The raw data are the measured time (*Time*), the machine displacement of the hydraulic jack (*Displacement*), the applied force (*Force*), the displacements at each LVDT (*LVDT1, LVDT2, LVDT3*), and the strains at the SGs (*SG1, SG2, SG3*). In addition, the absolute value of the machine displacement and the force are included, since a tensile force has been defined as positive within the machine, but an evaluation should be made as a result of a positive compressive force.

The ``*Imperfections FE*'' sheet shows the approximations for the numerical simulation of the measured imperfections via sine half-waves.

The data exported from the simulation (*LVDT_Komb.txt* and *Strain_Gauges_Komb.txt*) can be found in the spreadsheet ``*Data row FE*'', the data modified for the charts in ``*Data modified FE*'' and the charts for the comparison between numerical simulation and experimental test in the spreadsheets ``*Force-LVDT FE*'' and ``*Force-SG FE*''.

## 4 Experimental Design, Materials and Methods

### 4.1 Experimental studies

**Fig. 1:**
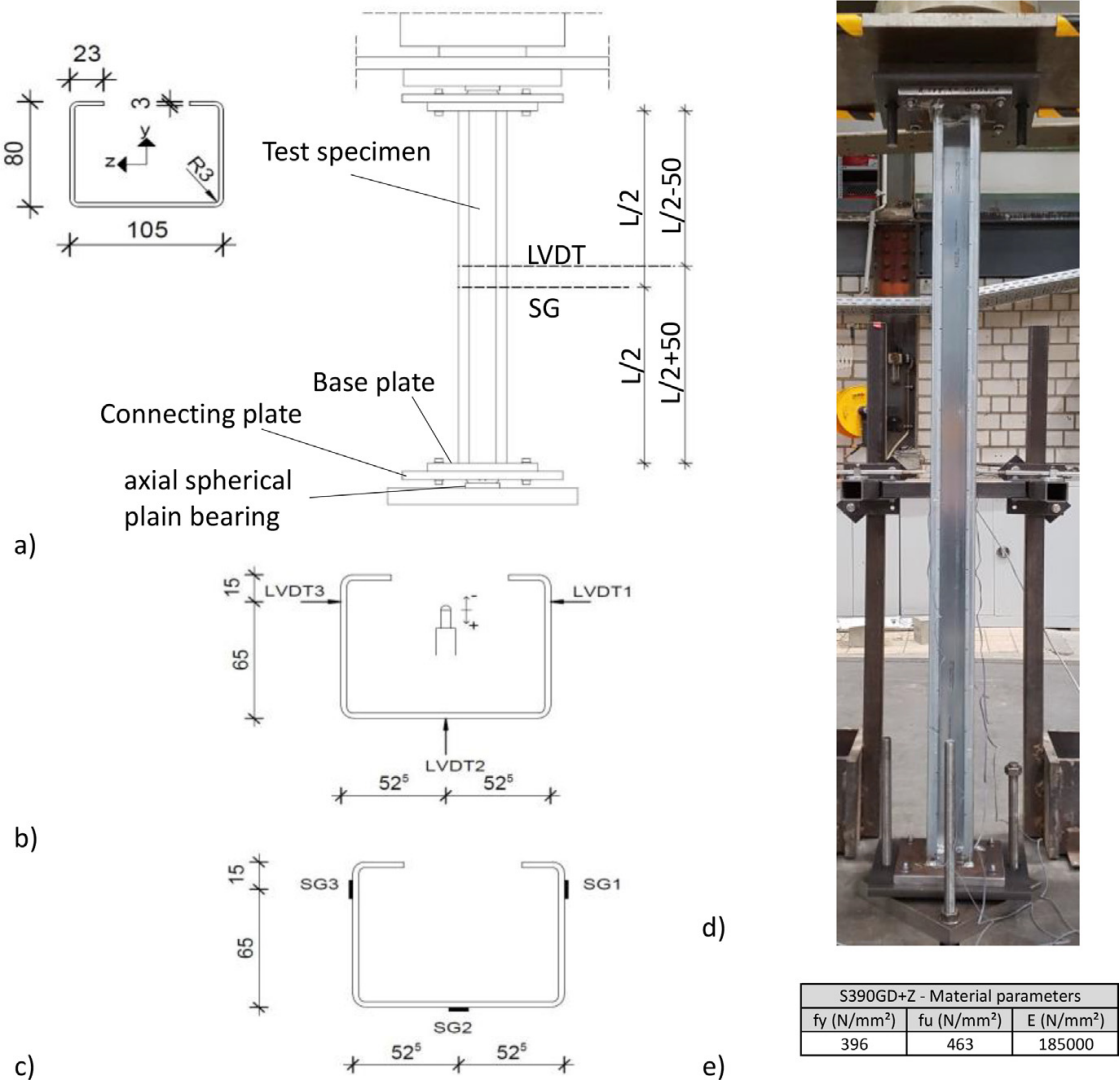
Test setup of the column compression tests a) Nominal dimensions of the C-shaped cross-section, sketch of the test setup and arrangement of the strain gauges (SG) and linear variable differential transformer (LVDT) over the test specimen length b) Arrangement of the LVDT over the cross-section c) Arrangement of the SG over the cross-section d) Photo of the test setup e) Material parameters.

The experimental data were generated as part of the FOSTA research project ``Future viability of cold-formed steel sections in building structures'' [2]. Three C-shaped thin-walled steel columns (material S390GD+Z, dimensions according to Fig. 1 a)) with a length of 1850 mm were investigated to determine the component load-bearing capacity of columns made of thin-walled cold-formed steel sections that are at risk of global stability.

The test setup was designed according to the specifications in EN 1993-1-3 [4] and EN 15512 [5] and is shown in Fig. 1 a) and d). The test specimens were welded at their ends with steel plates (depth 190 mm, width 240 mm, thickness 20 mm). These head plates were drilled ⌀=11 mm in the corners (each 25 mm from the outer edges). The simple support was generated using axial spherical plain bearings (GE 30 AW from Schaeffler Technologies). For this purpose, the head plates of the test specimens were each bolted to a connecting plate (250 mm, 350 mm, 20 mm), which had slotted holes (⌀=14 mm, L=25 mm) at the level of the head plate holes for alignment of the head plates. In addition, there was a threaded hole in the center of gravity of the connecting plate, into each of which the spherical part of the axial joint was inserted. The other part of the axial joint was attached to the upper end of the test specimen below the test jack and fixed to the base of the test specimen via a bearing plate supported in the clamping field. This generated a simple support of the specimen according to Euler buckling mode 2.

**Fig. 2:**
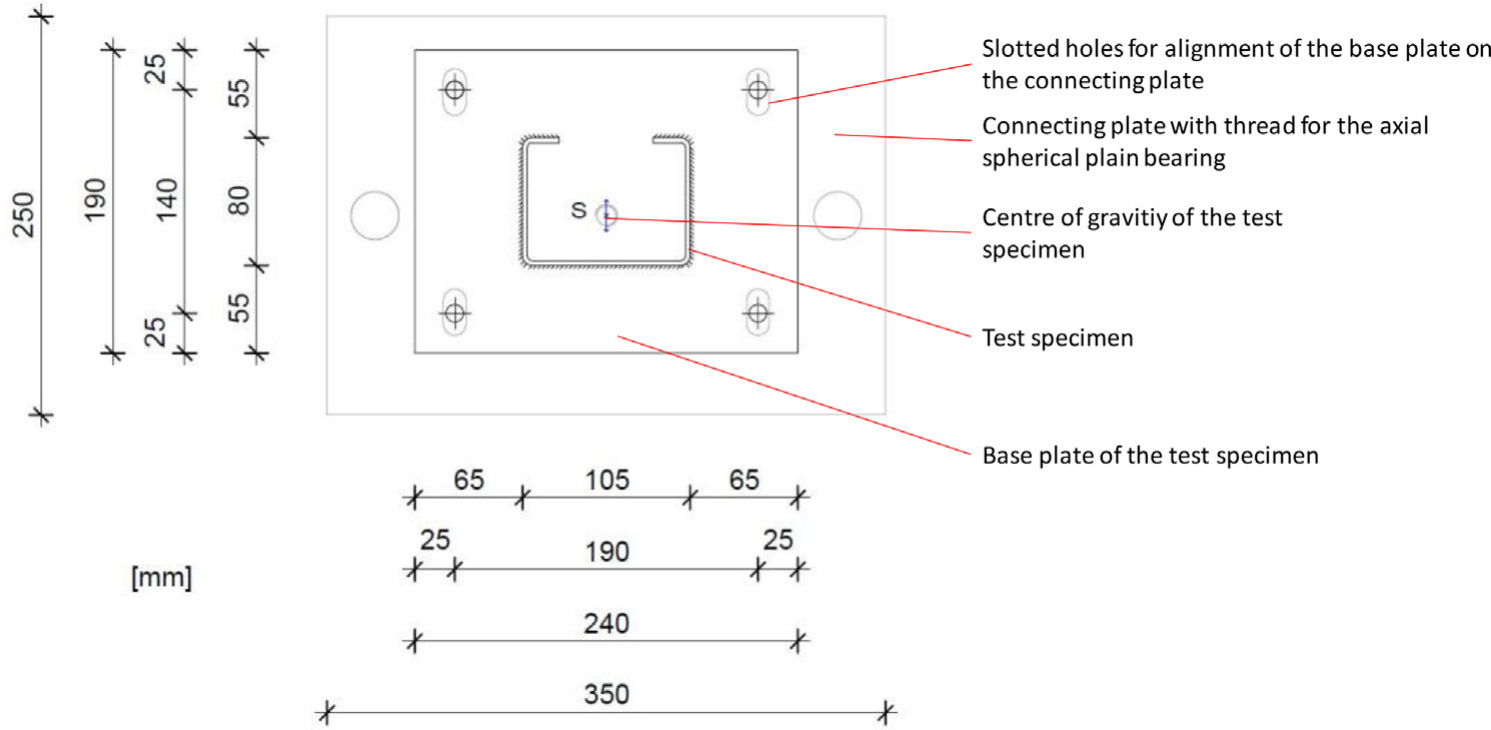
Connection of the base plate to the connecting plate.

Fig. 2 shows the connection of the base plate to the connecting plate.

The compression tests were carried out in a universal testing machine with a maximum cylinder compression force of 600 kN. Before the tests were carried out, the imperfections of the test specimens were measured at the cross-section and over the column length. First, the actual sheet thickness of the sections was determined at 8 points using digital calipers (accuracy 0.01 mm). The measurements were taken at the left and right lips at 225 mm, 725 mm, 1225 mm and 1725 mm from the column base respectively. Secondly, the opening dimension c of the section between the lips was determined, also using digital calipers. These measurements were made over the entire length of the specimen at a distance of 125 mm. In addition, a laser plane was set up parallel to the web and parallel to a flange, and the distance between the corners of the test specimen and the laser plane was determined by means of a ruler in order to be able to record the global precurvature.

**Fig. 3:**
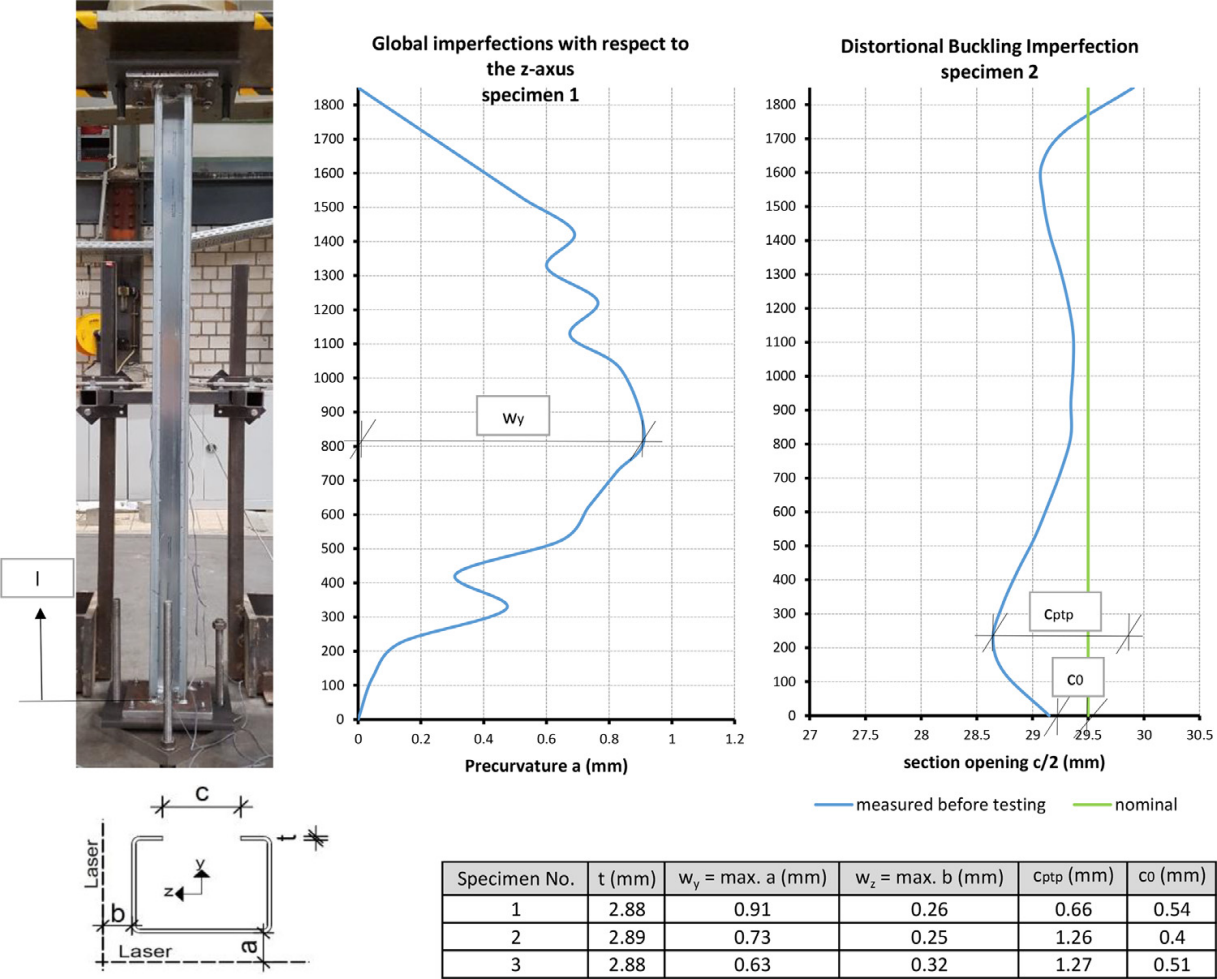
Definition of the measured imperfection values a, b, c, t and a compilation of the maximum amplitudes/values w_z_, w_y_, c_0_ and c_ptp_ and the mean values of the thicknesses t of the three investigated test specimens prior to the test procedure.

Fig. 3 is a compilation of the measured local imperfections on the steel cross-section and the global imperfections of the column over the column length of three test specimens before testing. The notation of the imperfections is explained in the diagrams. The measured sheet thickness t, the precurvature of the column in y-direction with the maximum value w_y_, the precurvature in z-direction with the maximum value w_z_ and the deviation of the lip position from the nominal value c/2 were documented. Two values were documented from c/2. First, the highest deviation of the lip position from the nominal value directly at the head plate c_0_. Secondly, the difference between maximum and minimum (peak-to-peak) of all measured values c_ptp_. The measured values at the individual measuring points as a function of the coordinate “l” can be taken from the Excel documents in the Folder “Experimental Data and Comparison to FE” for the respective test specimens in the spreadsheets ``*Imperfections*”.

The results of this measurement of the global precurvature were then converted, assuming that the base point and the head point of the test specimen were exactly above each other horizontally, so that an ideal precurvature was obtained with the zero crossings at the head and base points (see Excel files *Test Specimen 1.xlsx* to *Test Specimen 3.xlsx* spreadsheet ``*Imperfections*'').

Before the test specimens were installed in the test rig, three linear strain gauges (FLAB-6-11-3LJCT-F from TML) were applied in the center of the specimen. For this purpose, the zinc layer of the test specimens was removed, the corresponding areas were cleaned and the SG were glued on. This was done in the center of the web and at each flange 15 mm from the open edge, see Fig. 1c).

After the test specimens were installed in the rig, a tripod was set up on which the LVDTs (WA100 from HBM) could be aligned by means of magnetic holders. The LVDTs were mounted in a way that they were located at the same points in the cross-section as the SGs, but 50 mm above them, see Fig. 1 a) and c).

Both the SG and the LVDT were connected to a UPM 60 multi-point gauge, which collected the data and transferred it to a computer running data acquisition software CATMAN 6.0. In addition, the machine displacement and machine force from the machine controller were also transferred from the UPM to the computer running CATMAN software.

**Fig. 4:**
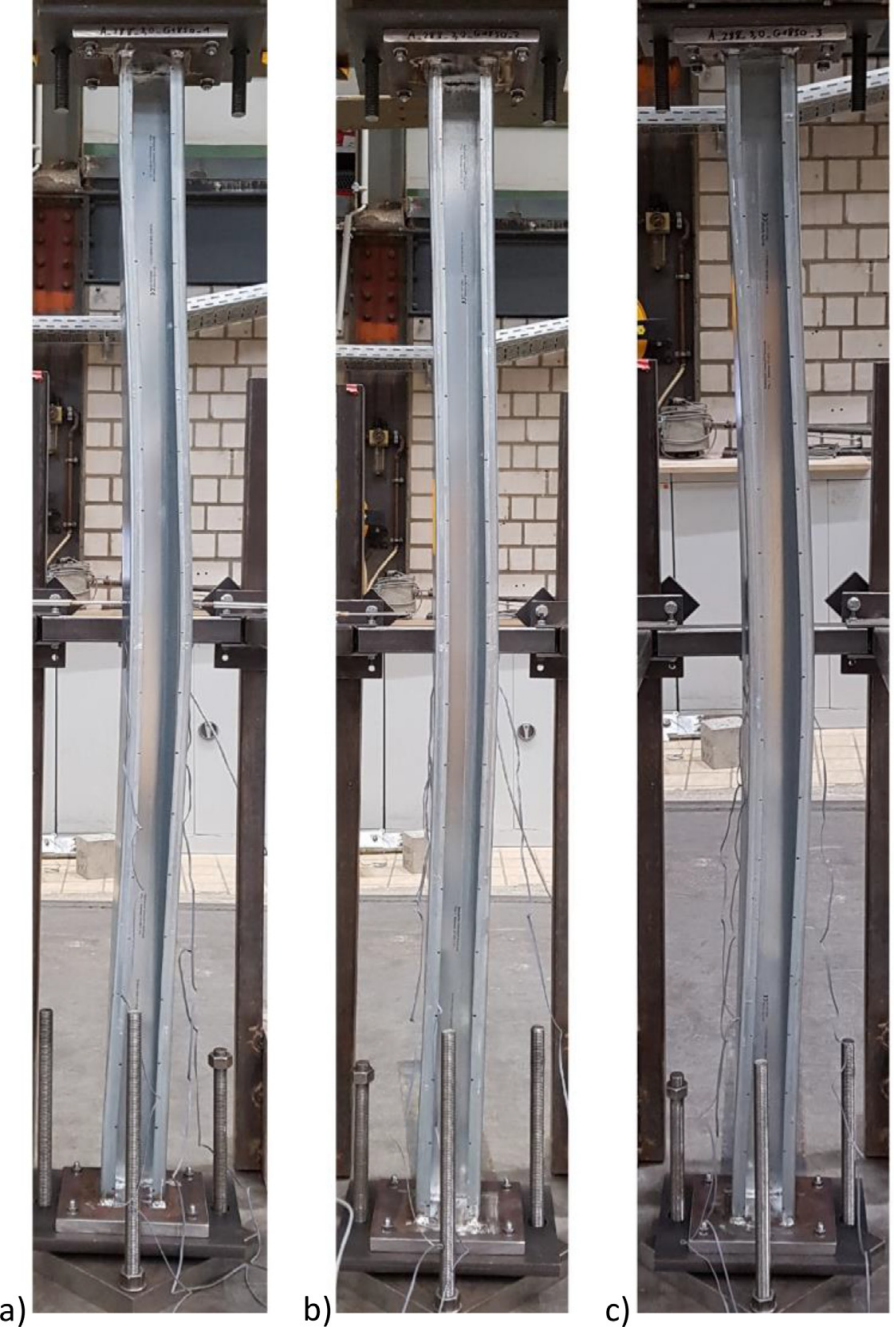
Deformation figures after performing the compression tests a) Test specimen 1 b) Test specimen 2 c) Test specimen 3.

The test load was applied displacement-controlled with a rate of 0.15 mm/min. The displacement control allowed the test specimen to be loaded beyond the maximum ultimate load so that the post load behavior could be investigated. Prior to the actual loading, the specimen was first subjected to a load of approx. 20 kN and then unloaded to a load of approx. 2 kN in order to exclude settlements in the test setup during the actual loading. After the load was exceeded, the machine displacement was further increased before unloading took place at a rate of 2.0 mm/min. The resulting failure mechanisms (flexural torsional buckling) were evidenced by photos taken by a smartphone (Samsung A41), see Fig. 4.

After unloading, the test specimens were removed and the measurement of the opening dimension c of the test specimens was repeated.

The data collected by CATMAN were exported as .txt and subsequently evaluated in Excel (see *Test Specimen 1.xlsx* to *Test Specimen 3.xlsx*, Sheet “*Data raw*” in the folder “Experimental Data and Comparison to FE”).

**Fig. 5:**
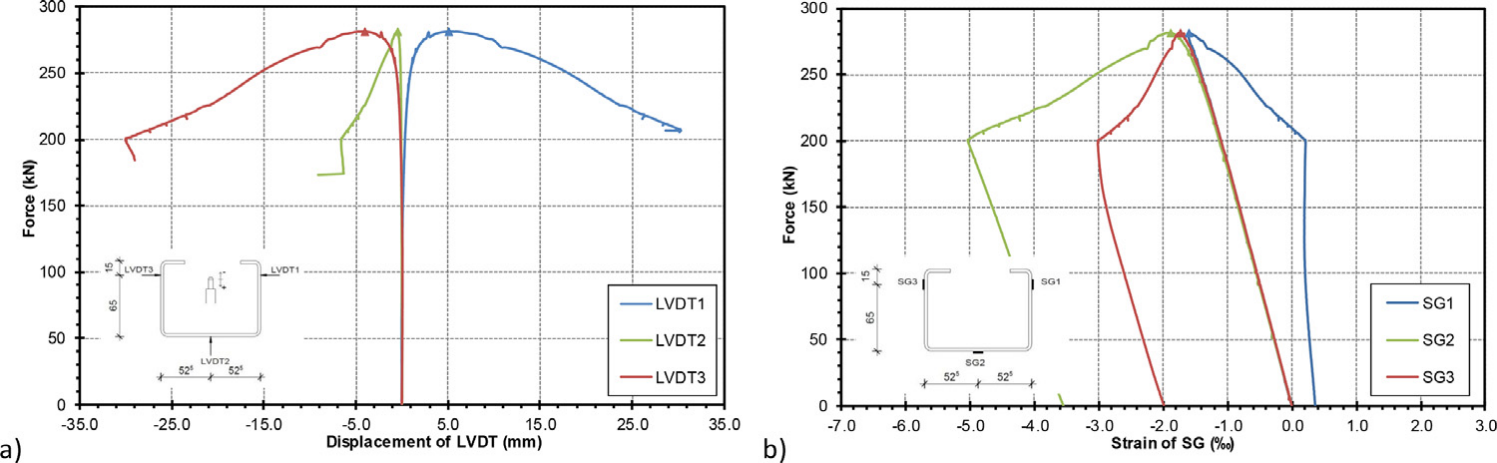
Measured values during the compression tests on Test Specimen 2 a) Measured deformations of the LVTD b) Measured strains of the SG.

The strains and stresses measured during the test are shown as an example for test specimen 2 in the diagrams in Fig. 5.

### 4.2 Numerical simulations

The numerical model was generated in the FE software ANSYS (2023 R1) via the program version ANSYS Classic. For this purpose, scripts were created in the program language ANSYS Parametric Design Language (APDL) (see *01_Buckling.mac, 02_Precurvature.mac, 03_DTB_Imperfection.mac, 04_Bearing_Load.mac* in the folder “FE input files”). The scripts were parameterized to compute different configurations, see Table 1.

**Fig. 6:**
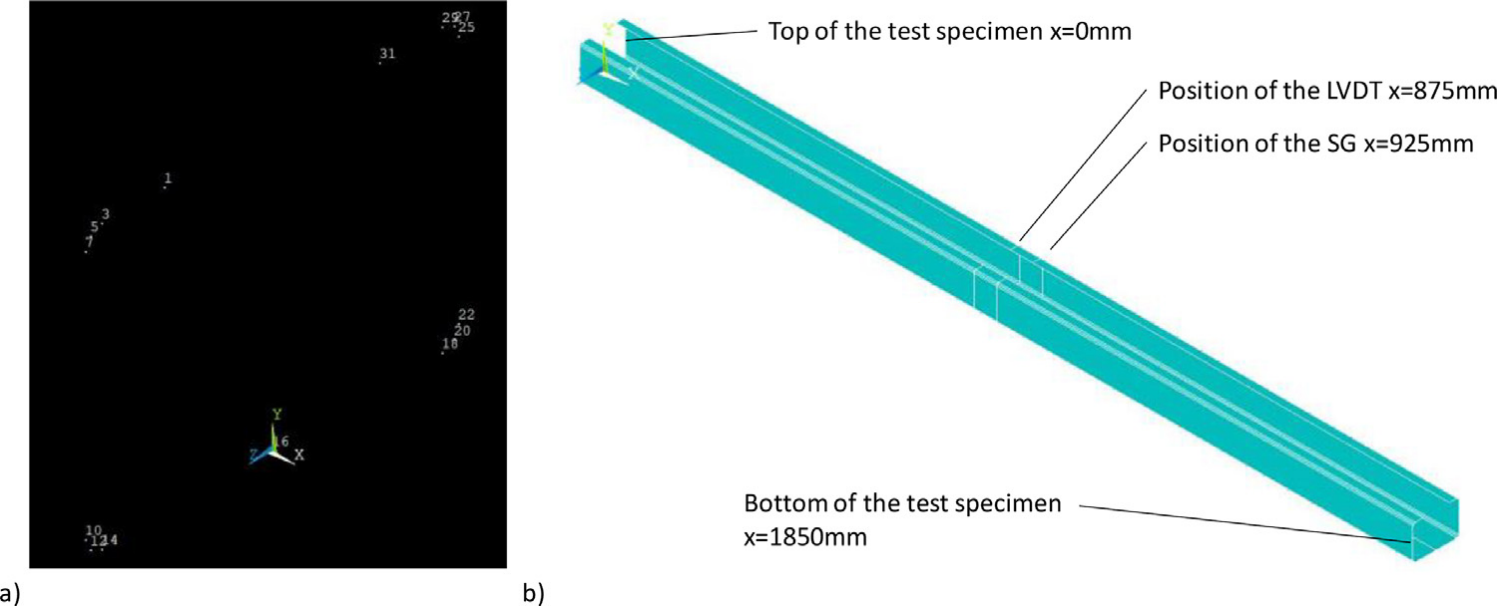
Generation of the numerical model a) Setting of keypoints at the upper end of the model (x=0mm) b) Geometry of the C-shaped section created form areas.

The geometry was created using keypoints at the ends of the test specimen, at the level of the strain gauges and the LVDTs in the real test, see Fig. 6 a). Subsequently, areas were generated from the keypoints (Fig. 6 b)), resulting in a shell model.

The head plates were generated analogously as areas.

According to EN 1993-1-14, Table 6.1, a simulation using the analysis type GMNIA includes both geometric and material non-linearity. In accordance with the recommendations of EN 1993-1-14, a simplified linear-elastic, ideal-plastic material model was used due to the expected global component failure. Material non-linearity is therefore a given.

**Fig. 7:**
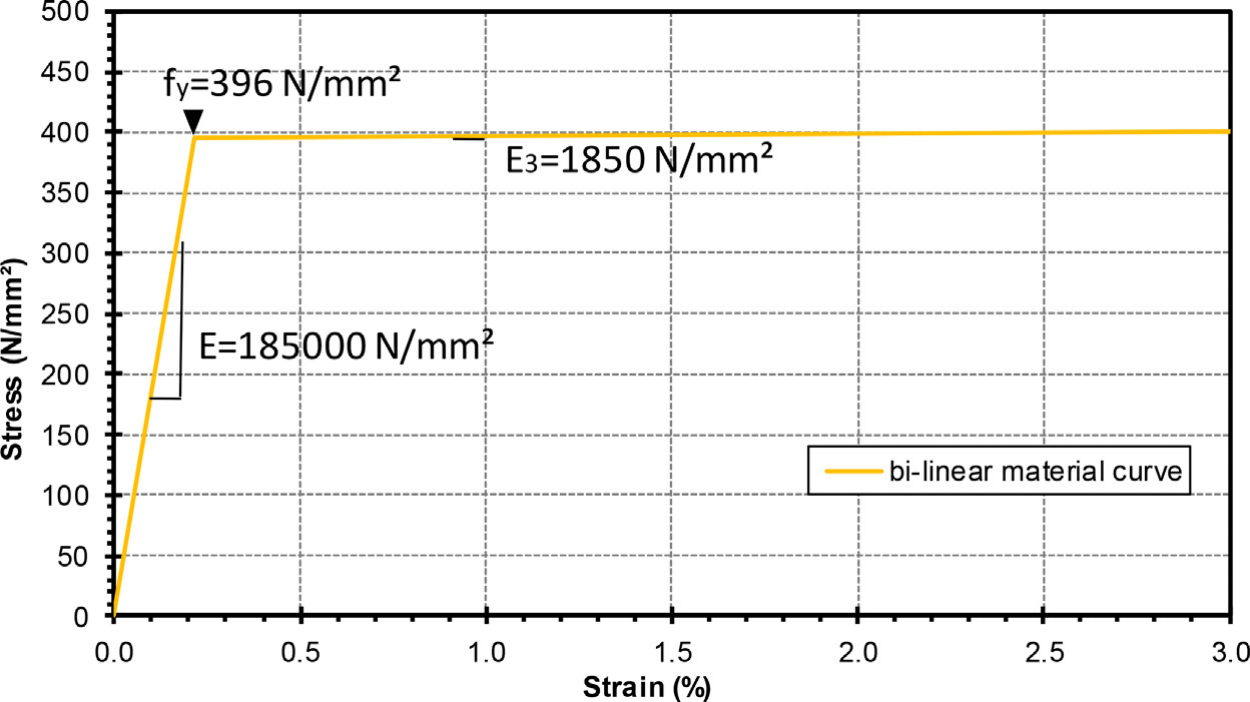
Material model for the nonlinear numerical calculations.

The value from the steel manufacturer's test certificate (f_y_=396 N/mm²), which was made available to the research institution, was used as the yield strength. The Young's modulus (185,000 N/mm²) was taken to be the value determined in the course of the research project from another batch of the same section by means of tensile tests in a Servo Hydraulic Universal Axial Testing Machine (PSA) in accordance with the specifications of DIN EN ISO 6892-1 [6]. The slope of the stress-strain diagram after reaching the yield point was set at E/100 according to the specifications of EN 1993-1-14, 5.3.3 (3), see Fig. 7.

Depending on the simulation, the meshing was done with elements of the type Shell 181 (linear approach, 4 nodes per element) or Shell 281 (quadratic approach, 8 nodes per element). Each node of the elements has 6 degrees of freedom (displacements in 3 directions, rotation around the 3 spatial axes).

**Fig. 8:**
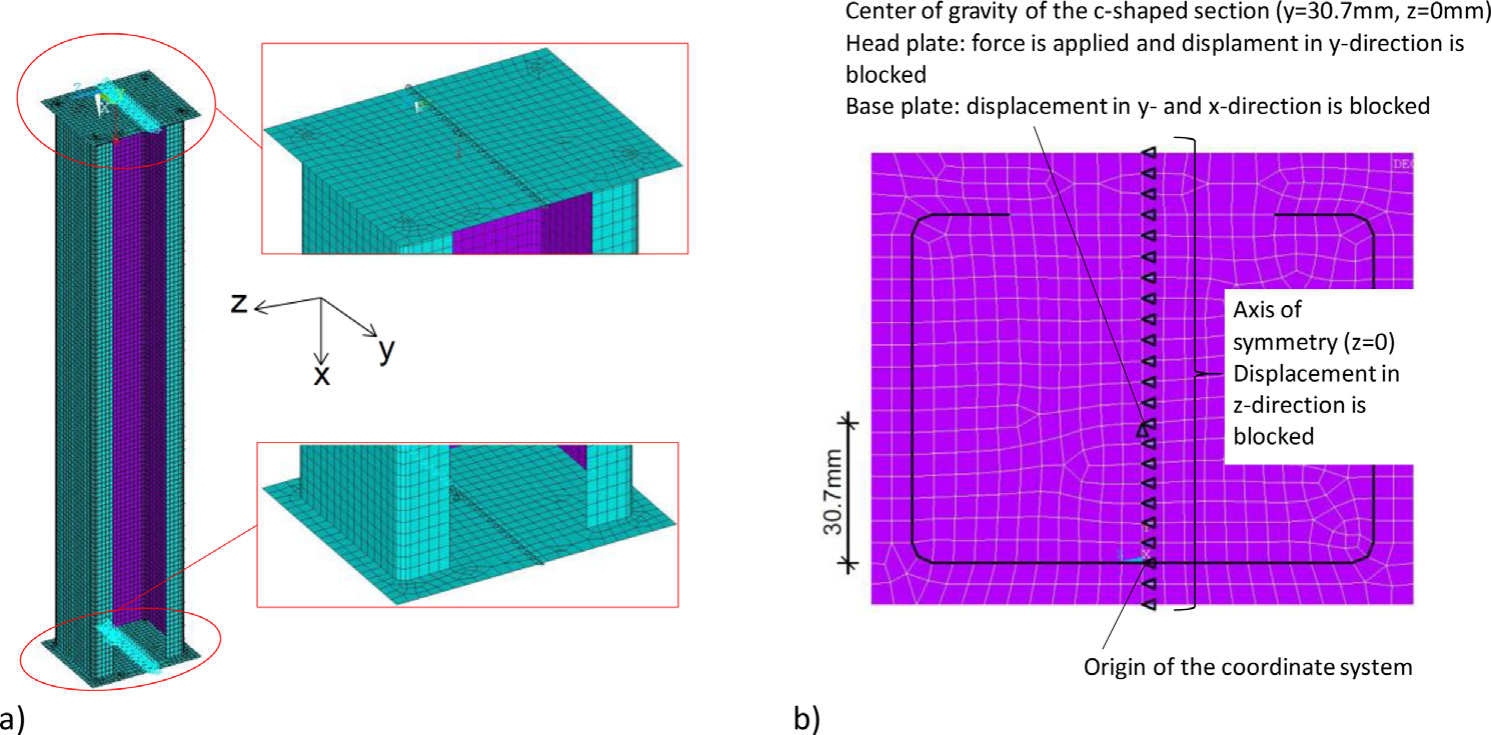
Numerical model and boundary conditions a) Overview b) detail of the base plate.

Fig. 8 shows the numerical model from ANSYS (2023 R1) with the boundary conditions used. The nodes of the head plates and the section were fused, resulting in a rigid connection that corresponds to the welded joint in the real test specimen.

The head plates were held in their axis of symmetry in the z-direction. At the center of gravity of the C-section, a concentrated force was applied to the head plate and the displacement in the y-direction was locked. At the base of the model, the corresponding node was supported in the y- and x-directions.

The geometric imperfections required for the analysis type GMNIA were used up during the development of the numerical model based on the specifications of EN 1993-1-14. When validating the model, however, the measured imperfections of the test specimens were also applied in accordance with Fig. 3.

Table 2 contains a compilation of the imperfections to be applied in the numerical model according to EN 1993-1-14, Clause 5.4.4 for compression-loaded, cold-formed steel sections with thin-walled cross-section corresponding to local buckling e_0,loc_ and distortional buckling e_0,dist_ as well as a global precurvature e_0,glob_ to represent global stability shapes. The section width b as nominal width b_p_ according to the specifications of EN 1993-1-3 Clause 5.1, the plastic axial force $N_{pl}=f_{y}\cdot A$ and the elastic critical buckling load for distortional buckling N_cr,d_ as well as the length L of the test specimens are included.

**Table 2:** Imperfections to be applied according to EN 1993-1-14, Clause 5.4.4.

Local Buckling			Distortional Buckling				Precurvature		
EC3-1-14	b (mm)	$e_{0,\mathrm{loc}}$ (mm)	EC3-1-14	$N_{\mathrm{pl}}$ (kN)	$N_{\mathrm{cr},d}$ (kN)	$e_{0,\mathrm{dist}}$ (mm)	EC3-1-14	L (mm)	$e_{0,\mathrm{glob}}$ (mm)
$\frac{b}{200}$	95.6	0.478	$0,3\cdot t\cdot \sqrt{\frac{N_{\mathrm{pl}}}{N_{\mathrm{cr},d}}}$	345.82	537.761	0.722	$\frac{L}{1000}$	1800	1.8

**Fig. 9:**
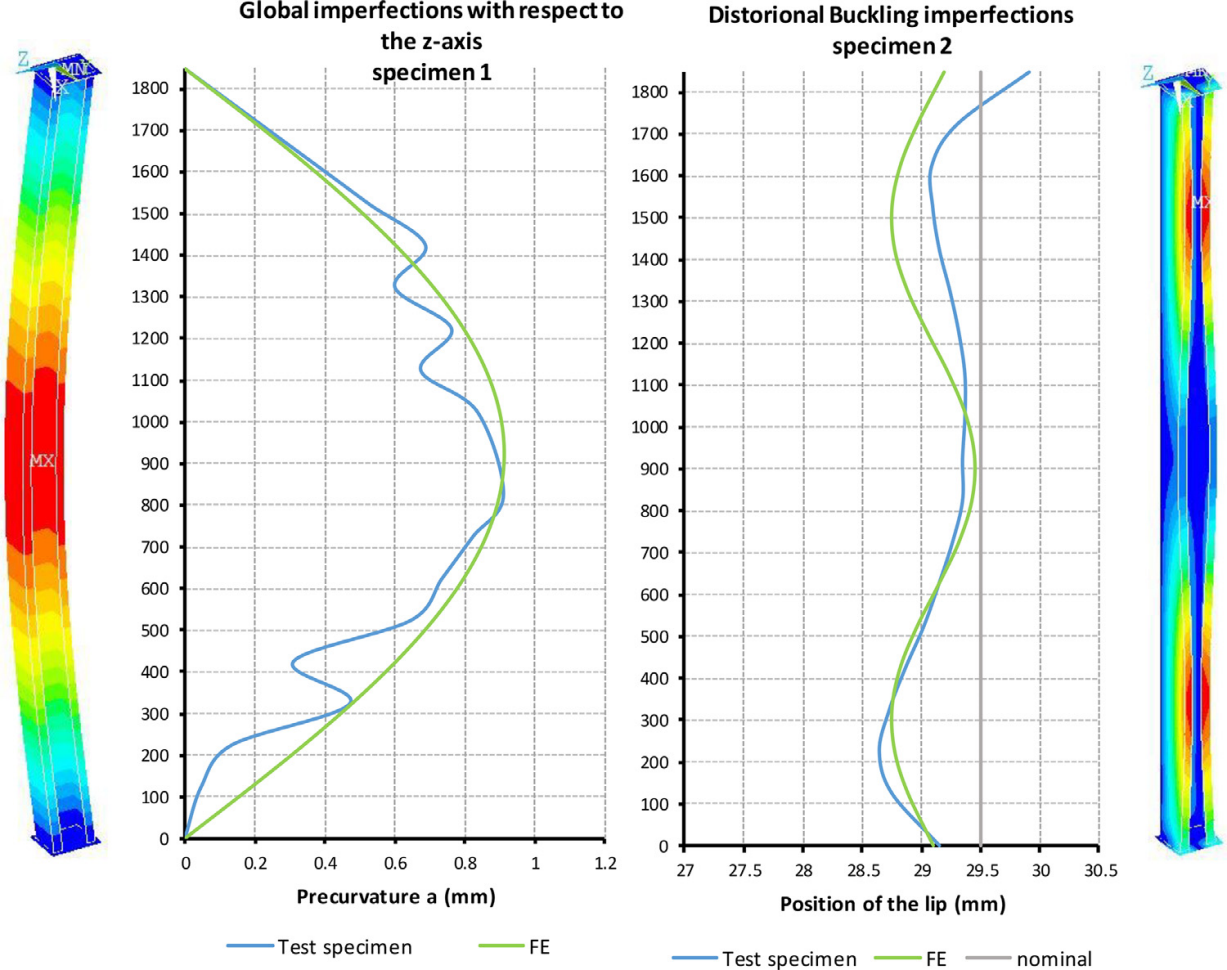
Approximation of the measured imperfections by sine half-waves for the numerical simulation.

The approximation of the imperfection shapes measured in the real test specimen via sine half-waves in the numerical model is shown in Fig. 9. The corresponding input data can be viewed in the Excel documents in the folder “Experimental Data and Comparison to FE” for the respective test specimen in the spreadsheet ``*Imperfections FE*''.

### 4.3 Sensitivity of mesh density

In order to minimize the time required for the calculation while at the same time ensuring the quality of the model, a sensitivity study of the element size and element type was started. The element size was varied between 1.25 and 40 mm. In addition, both Shell 181 and Shell 281 elements were considered.

Since the geometry was generated using manually set keypoints, the number of nodes in the rounded corners was also manually distinguished between no, one, or three intermediate nodes depending on the element size. Since the expected failure mode was known from the component tests (see Fig. 4), only a global precurvature in the z-direction, i.e. perpendicular to the symmetry axis of the cross-section, was applied in this study. Furthermore, in these initial studies, the material parameters were set according to Fig. 5, the nominal sheet thickness of the test specimens was chosen to be 3 mm, and the Newton-Raphson technique was selected for the calculation, since an evaluation of the obtained results was to be made using the maximum ultimate load. Since only the maximum ultimate load was compared in all parameter studies, the use of the Newton-Raphson technique is sufficient, as the behavior of the component is irrelevant when the load increases beyond the ultimate load. The full Newton-Raphson technique was used, the load was ramped to 500 kN and 500 substeps were allowed.

For the generation of the results, the calculation *02_Precurvature.mac* was first performed with the appropriate inputs for the size of elements, the element type, the sheet thickness t=3.0 mm and the precurvature *Vari_Prec* 1 (length/1000 in z-direction). In this routine, a new position is assigned to each node of the model based on a sine half-wave, so that a uniform precurvature is created, analogously to Fig. 9.

Subsequently, *04_Bearing_load.mac* was applied with the same input parameters (element type, element size, t=3.0 mm) as in the previous simulation of the precurvature, so that the simulation could take place with consideration of the precurvature. *04_Bearing_load.mac* accesses thereby the results of *02_Precurvature.mac* and updates the node position in the numerical model before the execution of the calculation. Only the coordinates of the nodes are transferred. Stresses and strains are not considered.

Table 3 summarizes the results of the numerical study with different element types and mesh densities. The table differentiates the element size *etG*, the number of degrees of freedom (DOF) and the numerically obtained ultimate load R_FE_. In addition, an extrapolated limit value of the mesh density study is included for each element type. The results of the simulations in Table 3 can be found in the folder ``FE Data'' in the txt files Strain_Gauges_13_*etG*_*etTyp.txt* (the maximum load R_FE_ can be found in the last line of the second column (*Machine-Force*) in MN) and in the Excel file *FE_Output.xlsx*.

**Table 3:** Maximum ultimate load for different element types and sizes.

Element type *etTyp*	Element size (*etG*) (mm)	Number of DOF	R_FE_ (kN)
Linear (ANSYS SHELL 181)	40	4926	267.79
	20	12762	267.75
	10	44658	268.04
	5	151056	270.32
	2.5	580422	271.52
	1.25	2289852	270.96
		Extrapolated value	271.33

Quadratic (ANSYS SHELL 281)	40	14478	268.07
	20	37698	267.56
	10	115296	267.81
	5	450816	270.60
	2.5	1736502	270.75
	1.25	6860022	270.90
		Extrapolated value	270.86

**Fig. 10:**
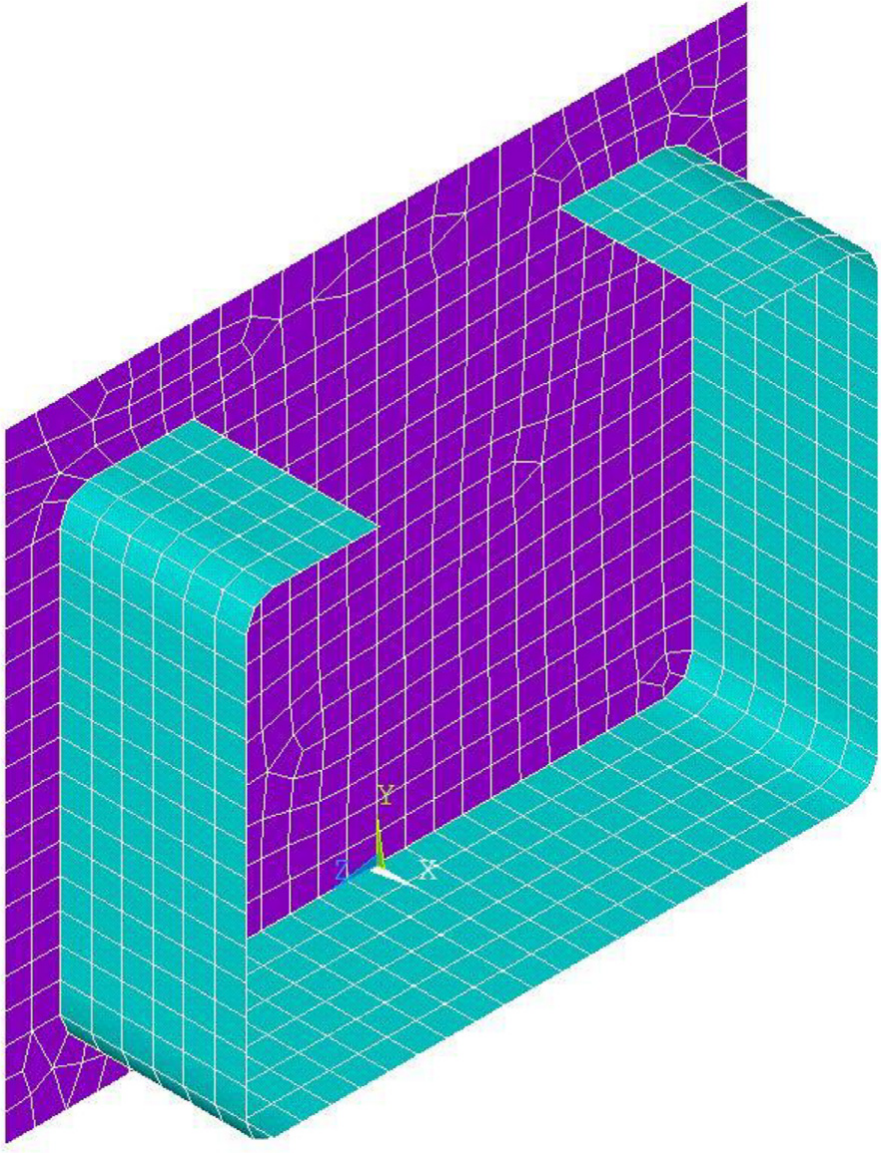
Selected mesh density for the further simulations (*etG*=5, Shell 181).

**Fig. 11:**
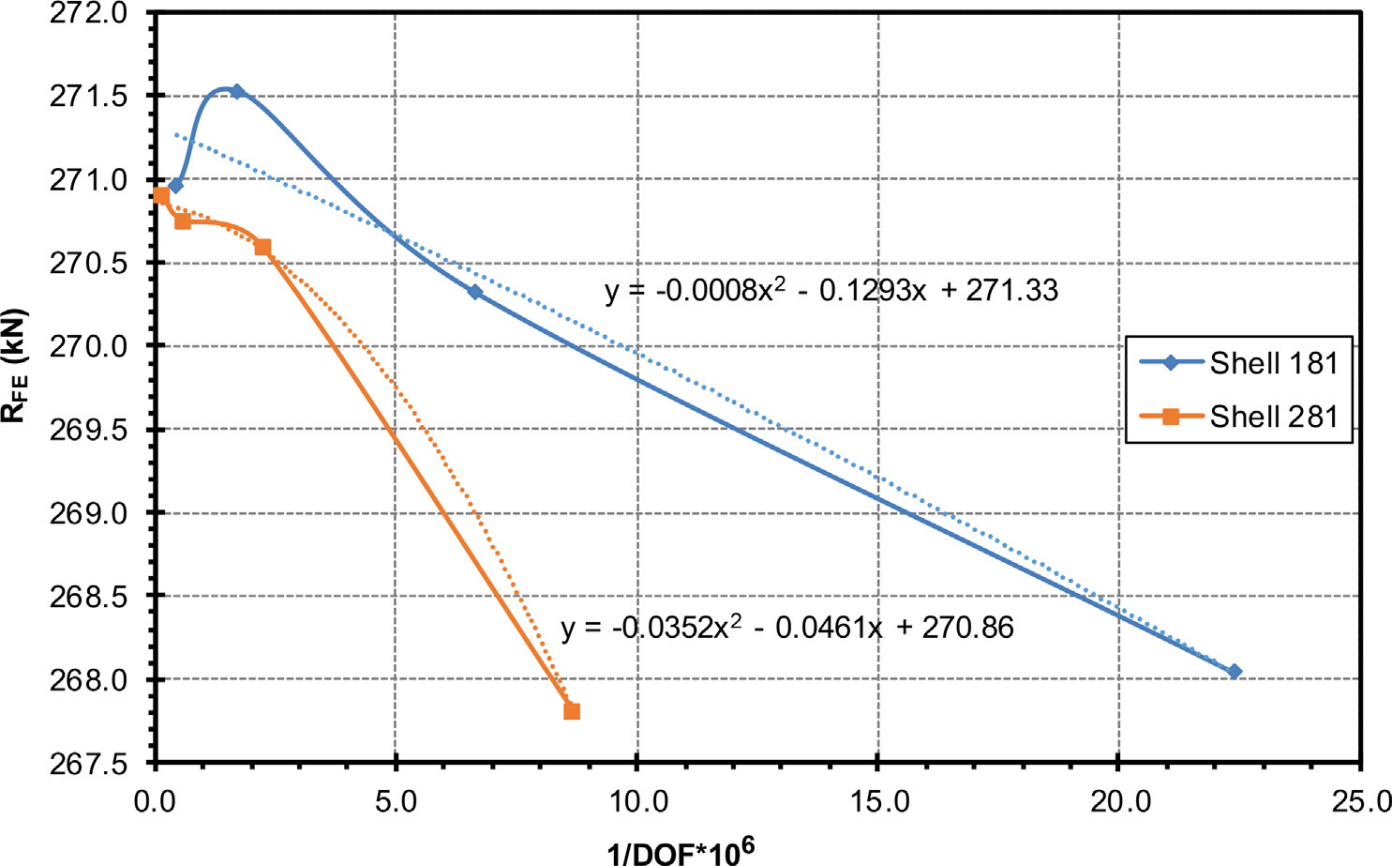
Mesh convergence study.

The generated results of the sensitivity study were evaluated in terms of their quality in comparison to a limit value extrapolated by means of Excel (quadratic approach, see Fig. 11) and the required calculation effort. For the further investigations, Shell 181 in an element size of 5 mm was selected as the element type (Fig. 10), since the deviation from the extrapolated value is only 0.3 %.

### 4.4 Sensitivity of imperfections

In order to record the sensitivity of the model to various imperfections, the amplitude magnitude of the imperfections was first determined according to EN 1993-1-14 (Table 2) and applied either as a sine half-wave or via the previously performed elastic critical buckling analysis (Fig. 9).

In the study of sensitivity to local imperfection of the cross-section corresponding to local buckling, an elastic critical buckling analysis was performed using routine *01_Buckling.mac*. In this routine, the 25 first eigenvalues were determined for the component under axial compression load. The Eigen modes were evaluated by visual analysis considering the failure mechanism. The first three Eigen figures (lowest eigenvalues), which could be clearly assigned to the local buckling (eigenvalue 5-7), were selected for the study. In contrast to Fig. 7, a linear-elastic material model with E=185,000 N/mm² is used in *01_Buckling.mac.*

The numerical load calculation is then performed using routine *04_Bearing_load.mac*. For this study (Table 4), 6 calculations were performed which executed the imperfection combinations *Komb* 1 to *Komb* 6. These combinations included the three previously determined local Eigen modes each scaled positively (in the direction of the Eigen mode) and negatively (inversely to the Eigen mode) to the normative value given in Table 2. For this purpose, the component geometry was updated after the model was generated, before the calculation was performed, so that the stress-free geometry was created corresponding to the deformed component's eigenvalue.

**Table 4:** Sensitivity to local imperfection corresponding to local buckling and assignment to the imperfection combinations *Komb* in the input files (mac).

Direction	R_dM5_ (kN)	R_dM6_ (kN)	R_dM7_ (kN)	R_dM5_/R_dMin_	R_dM6_/R_dMin_	R_dM7_/R_dmin_
Positive	301.82 (*Komb* 1)	301.76 (*Komb* 3)	306.24 (*Komb* 5)	1.00	1.00	1.02
Negative	301.20 (*Komb* 2)	301.34 (*Komb* 4)	307.36 (*Komb* 6)	1.00	1.00	1.02
Neg/Pos	1.00	1.00	1.00			

The procedure for generating the data for sensitivity analysis with respect to an imperfection corresponding to distortional buckling (Table 5) was carried out analogously. From the 25 Eigen modes of a calculation according to *01_Buckling.mac*, the first 3, which could be clearly assigned to the stability phenomenon distortional buckling (eigenvalue 3-4, 19), were applied both positively and negatively for the calculation of the ultimate load (carried out with *04_Bearing_Load.mac* using the imperfection combinations *Komb* 7-12).

**Table 5:** Sensitivity to cross-sectional imperfection corresponding to distortional buckling and assignment to the imperfection combinations *Komb* in the input files (mac).

Direction	R_dM3_ (kN)	R_dM4_ (kN)	R_dM19_ (kN)	R_dM3_/R_dMin_	R_dM4_/R_dMin_	R_dM319_/R_dmin_
Positive	301.88 (*Komb* 7)	300.18 (*Komb* 9)	318.78 (*Komb* 11)	1.02	1.01	1.07
Negative	296.64 (*Komb* 8)	301.98 (*Komb* 10)	319.43 (*Komb* 12)	1.00	1.02	1.08
Neg/Pos	0.98	1.01	1.00			

The first (smallest) eigenvalue for distortional buckling N_cr,d_ from the buckling analysis in ANSYS was applied to calculate the amplitude to be applied according to EN 1993-1-14, 5.4.4 (8) (see Table 1), so that all 6 simulations in this study were performed based on the same amplitude of imperfection but different imperfection shapes.

The third sensitivity study dealt with the sensitivity to a global precurvature. For this purpose, the routine *02_Precurvature.mac* was executed, in which either a sinusoidal precurvature in z- or in positive or negative y-direction is generated. In routine *04_Bearing_load.mac*, the results of *02_Precurvature.mac* were also applied as an imperfection by uploading the geometry between model generation and calculation (the combination *Komb* 13-15 was applied for this purpose) and thus the maximum attainable ultimate load was calculated (Table 6).

**Table 6:** Sensitivity to a global precurvature and assignment to the imperfection combinations *Komb* in the input files (mac).

Direction	R_dM_ (kN)	R_dM_/R_dmin_
z	270.32 (*Komb* 13)	1.00
y negative	271.58 (*Komb* 14)	1.00
y positive	286.30 (*Komb* 15)	1.06

Table 4, Table 5, Table 6 summarize the results of sensitivity studies regarding different imperfection approaches corresponding to local buckling and distortional buckling as well as a global precurvature as initiator of possible global instability shapes. In each case, the maximum load R_dMi_ of the simulation using different Eigen modes (associated to eigenvalue number i) as imperfection and the ratio of actual value i and minimum load of the study R_dmi_/R_dMin_ are given. The maximum load R_dmi_ for all simulation of the Table 4, Table 5, Table 6, Table 7, Table 8 can be found in the folder “FE Data” in the Excel file *FE Output.xlsx*.

**Table 7:** Sensitivity of the numerical simulation at different combinations of imperfections.

No.	Global imperfection z-direction	Global imperfections negative y-direction	Local Buckling Direction and combination factor	Distortional Buckling Direction and combination factor	R_dM_ (kN)	Percentage deviation to *Komb* 13
*Komb* 13	Yes	No	0	0	270.32	0.00%
*Komb* 14	No	Yes	0	0	271.58	0.47%
*Komb* 16	Yes	No	1	0	266.13	-1.55%
*Komb* 17	Yes	No	−1	0	265.92	-1.63%
*Komb* 18	Yes	No	1	1	269.63	-0.26%
*Komb* 19	Yes	No	−1	1	270.39	0.02%
*Komb* 20	Yes	No	1	−1	261.04	-3.44%
*Komb* 21	Yes	No	−1	−1	260.84	-3.51%
*Komb* 22	Yes	No	0	−1	260.73	-3.55%
*Komb* 23	Yes	No	0	1	275.34	1.85%
*Komb* 24	No	Yes	1	0	271.86	0.57%
*Komb* 25	No	Yes	−1	0	271.97	0.61%
*Komb* 26	No	Yes	1	1	260.57	-3.61%
*Komb* 27	No	Yes	−1	1	260.02	-3.81%
*Komb* 28	No	Yes	1	−1	249.52	-7.70%
*Komb* 29	No	Yes	−1	−1	253.23	-6.32%
*Komb* 30	No	Yes	0	−1	248.43	-8.10%
*Komb* 31	No	Yes	0	1	258.23	-4.47%

In a fourth study on the sensitivity of the numerical model to imperfection combinations, 16 further simulations were performed with different combined imperfection approaches (*Komb* 16-31). For this purpose, when updating the geometry in the routine *04_Bearing_load.mac*, both an imperfection from a previous calculation from *02_Precurvature.mac* and one or two imperfections from *01_Buckling.mac* (imperfection corresponding to local buckling and/or distortional buckling) were used. As it was shown in the Tables 4 and 5, the sensitivity to the imperfections corresponding to the local buckling and the distortional buckling is low. Only the Eigen mode of the lowest eigenvalue, which could be clearly assigned to the local buckling (eigenvalue 5) or the distortional buckling (eigenvalue 3), was considered in the further simulation.

A compilation of the numerically determined ultimate loads R_dM_ for the nominal section thickness t=3.0 mm with different combinations of the various imperfection approaches can be found in Table 7. Each combination is assigned which imperfections were applied in which orientation. In addition, the table includes a comparison between the ultimate loads of each combination to the combination *Komb* 13, since this combination includes only the imperfection corresponding to the stability failure observed in the tests. The R_dM_ values can be found in the repository in the folder “FE Data” in the corresponding sheets of the respective imperfection combination *Komb* in the Excel file *FE_Output.xlsx*.

### 4.5 Validation

According to the specifications of EN 1993-1-14, the imperfection combinations that result in the lowest ultimate loads in the numerical simulation are generally to be selected.

The comparison between test results and numerical simulation was therefore based on selected, unfavorable imperfection combinations from Table 7. The combinations *Komb* 13 (decisive imperfection for the instability of torsional flexural buckling that occurred in the test), *Komb* 22 (lowest ultimate load when applying a precurvature in the z-direction) and *Komb* 30 (absolute minimum of the ultimate loads from Table 7) were selected. The simulations with *Komb* 13, 22 and 30 were performed with the nominal value of the sheet thickness t=3.0 mm. Since the actual measured thickness of the test specimens was used for the following simulations (according to Fig. 3), this results in the new combination numbers *Komb* 32-40.

**Fig. 12:**
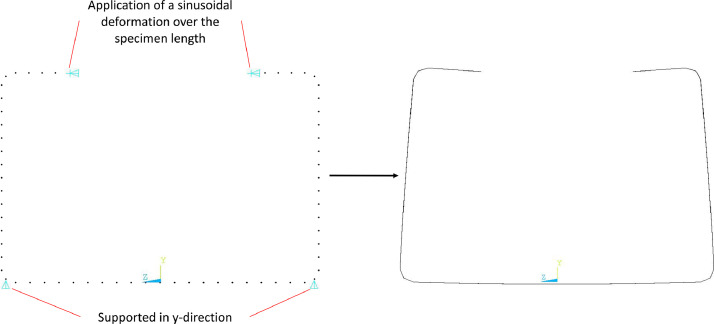
Boundary conditions for generating a DTB-Imperfection and deformed C-shaped section.

In addition, simulations were performed using the imperfections measured in the test. For this purpose, the routine *02_Precurvature.mac* was used to generate precurvatures around the y- and z-axis according to the measured amplitudes (see Fig. 3). On the other hand, imperfections corresponding to distortional buckling was applied via routine *03_DTB_Imperfection.mac*. While in the case of precurvature, each node of the model was assigned a new position corresponding to the sine half-wave, the creation of the DTB imperfection is only done by a sinusoidal displacement of the outermost nodes in the lips of the cross-section. At the same time, the nodes in the middle of the corners were supported in a non-displaceable manner in y-direction, see Fig. 12, so that the position of the component is preserved, while only a change in the opening dimension could be created (see also Fig. 9).

In all simulations of this last study, the solution algorithm was changed from the Newton-Raphson technique (*Sol_Meth*=1 according to Table 1) to the arc length technique (*Sol_Meth*=2 according to Table 1) in order to be able to analyze the bearing behavior beyond the maximum ultimate load and to grant a better comparison between test and numerical simulation. The standard ANSYS settings were used: a maximum radius multiplier of 25 and a minimum radius multiplier of 1/1000.

A comparison between test results and numerically determined ultimate loads using the measured sheet thicknesses of the test specimens and different imperfection combinations according to EN 1993-1-14 in comparison with the approach of the imperfections measured in the test is shown in Table 8. R_FE,test_ represents the ultimate load using the imperfections measured in the test. R_FEi_ is the ultimate load using the normative imperfections (*Komb* 13, 22, 30 from Table 7) and the sheet thickness t measured on the test specimen. The thickness-dependent amplitude of the imperfection corresponding to the distortional buckling e_0,dist_ is also given. A comparison between the ultimate loads R_test_ achieved in the test to the various numerically determined ultimate loads is also included. The values of R_FE_ can be found in the repository in the folder “FE Data” in the corresponding sheets of the respective combination *Komb* in the *Excel file FE_Output.xlsx*.

**Table 8:** Comparison between results of numerical simulation and ultimate loads of component tests under approach of different imperfections.

Test No.	t (mm)	e_0,dist_ (mm)	R_test_ (kN)	R_FE13_ (kN)	R_FE22_ (kN)	R_FE30_ (kN)	R_FE,test_ (kN)	R_FE1_ R_test_	R_FE22_ R_test_	R_FE30_ R_test_	R_FE,test_ R_test_
1	2.88	0.707	277.6	258.76 *Komb* 32	247.65 *Komb* 33	236.71 *Komb* 34	289.15 *Komb* 41	0.93	0.89	0.85	1.04
2	2.89	0.708	281.5	259.68 *Komb* 35	248.59 *Komb* 36	237.78 *Komb* 37	295.76 *Komb* 42	0.92	0.88	0.84	1.05
3	2.88	0.707	289.9	258.76 *Komb* 38	247.65 *Komb* 39	236.71 *Komb* 40	294.15 *Komb* 43	0.89	0.85	0.81	1.01

**Fig. 13:**
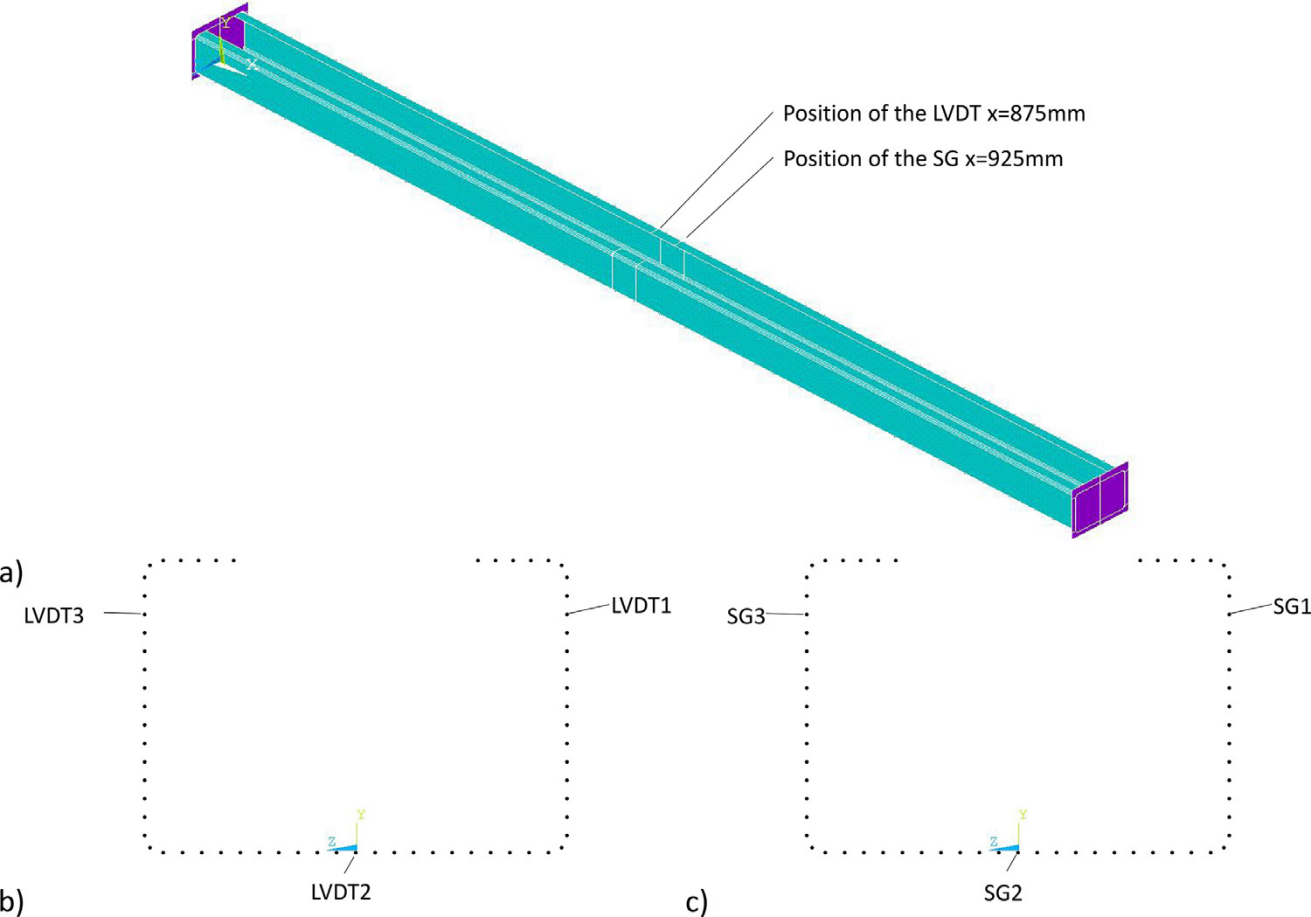
Position of the nodes at which the deformations or stresses and strains were evaluated a) Sections on which the nodes are located (corresponding to the LVDT and SG) b) Nodes at the positions of the LVDT c) Nodes at the positions of the SG.

*04_Bearing_Load.mac* contains additionally a routine, that two .txt files per calculation for separate analysis of deformations and strains are output *LVDT_Komb.txt* and *Strain_Gauges_Komb.txt* (with the used combination *Komb*). These data were then inserted into the ``*Data row FE*'' spreadsheets and Excel was used to set up a comparison between the deformations and strains from the numerical simulations and the component tests. Fig. 13 shows the positions of the nodes at which the displacements or the stresses and strains from the numerical model were recorded. Table 9 summarized the coordinates of the nodes and the recorded values.

**Table 9:** Coordinates of the nodes from Fig. 12 and the data extracted in each case.

node	x (mm)	y (mm)	z (mm)	Extracted data
LVDT1	875	63.786	-51	*South_Displacement* (Displacement in z-direction)
LVDT2	875	0	0	*East_Displacement* (Displacement in y-direction)
LVDT3	875	63.786	51	*North_Displacement* (Displacement in z-direction)
SG1	925	63.786	-51	*South_Strain, South_Stress* (Strain and Stress in x-direction)
SG2	925	0	0	*East_Strain, East_Stress* (Strain and Stress in x-direction)
SG3	925	63.786	51	*North_Strain, North_Stress* (Strain and Stress in x-direction)

**Fig. 14:**
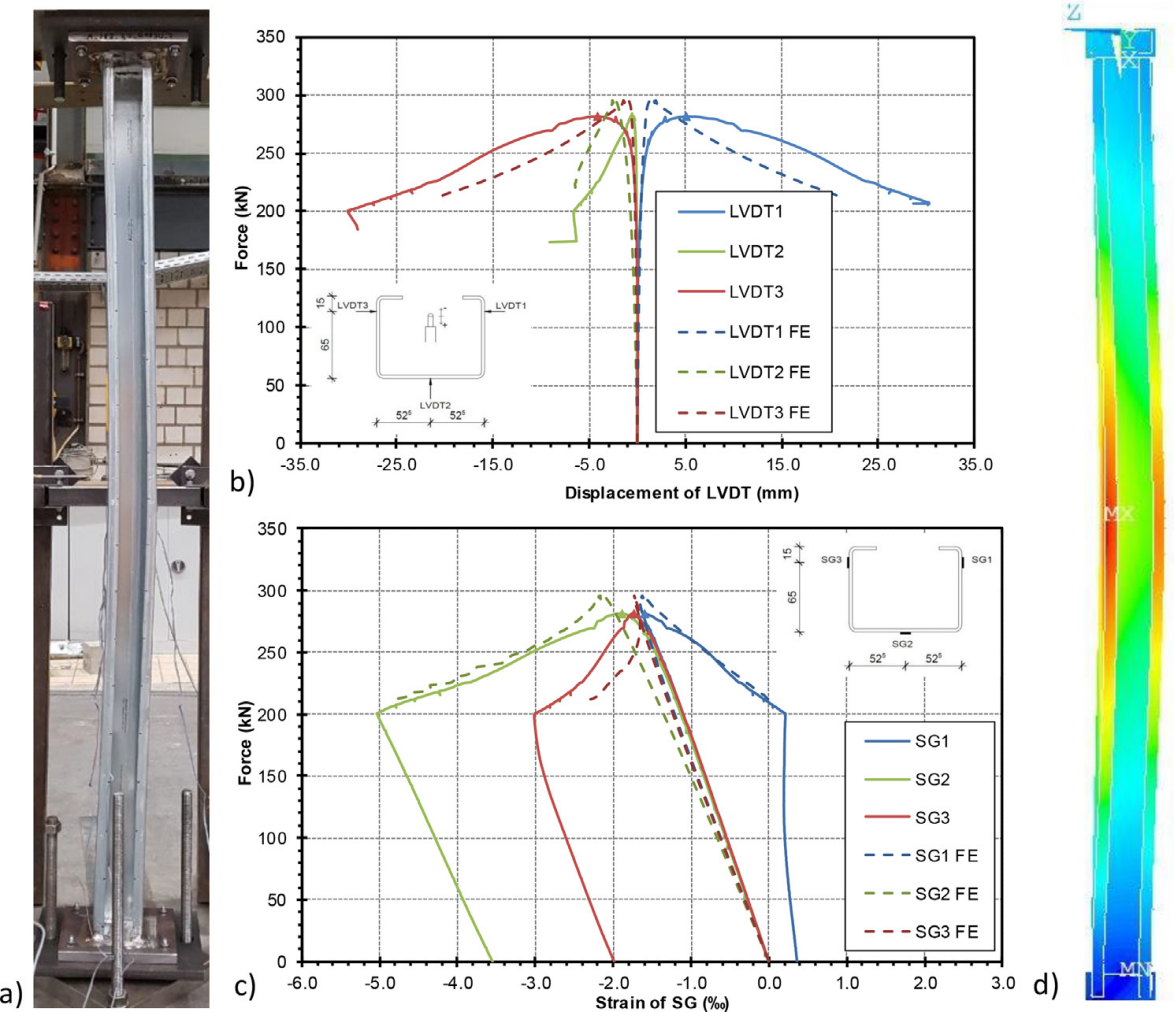
Comparison between numerically determined deformations and strains and the values measured by the SG and LVDT during the test a) experimental failure mode (specimen 2) b) comparison between deformations c) comparison between strains d) numerical failure mode.

A comparison between the deformations and the strains of the specimen measured in the test and the numerically determined values using the imperfection combinations *Komb* 42 as well as the corresponding deformation figures after the end of the load can be taken from Fig. 14 as an example for test specimen 2. For each of the three test specimens, the comparisons between test results and numerical simulation can be taken from the Excel files *Test Specimen 1.xlsx* to *Test Specimen 3.xlsx* in the sheets ``*Force-LVDT*'' and ``*Force-SG*'' in the folder “Experimental Data and Comparison to FE”.

## Limitations

Not applicable.

## Ethics Statement

The authors confirm that they have read and follow the ethical requirements for publication in Data in Brief and confirm that the current work does not involve human subjects, animal experiments, or any data collected from social media platforms.

## CRediT authorship contribution statement

**Dieter Ungermann:** Conceptualization, Methodology, Resources. **Tim Lemański:** Methodology, Formal analysis, Investigation, Data curation, Writing – original draft, Visualization. **Bettina Brune:** Conceptualization, Methodology, Validation, Writing – review & editing, Funding acquisition, Visualization, Supervision, Project administration, Funding acquisition.

## Data Availability

Dataset of a numerical model of a cold-formed, C-shaped section in compression according to EN 1993-1-14 (Original data) (Mendeley Data)
